# Cortical representation of experimental periodontal pain: a functional magnetic resonance imaging study

**DOI:** 10.1038/s41598-021-94775-4

**Published:** 2021-08-03

**Authors:** Angelika Maurer, Damian Verma, Annika Reddehase, Lukas Scheef, Alexander Radbruch, Ulrike Attenberger, Andreas Jäger, Henning Boecker

**Affiliations:** 1 Functional Neuroimaging Lab, Department of Diagnostic and Interventional Radiology, University Hospital Bonn, Bonn, Germany; 2 Department for Orthodontics, University Hospital Bonn, Bonn, Germany; 3 Department of Neuroradiology, University Hospital Bonn, Bonn, Germany; 4 Department of Diagnostic and Interventional Radiology, University Hospital Bonn, Bonn, Germany; 5 Chair of Functional Neuroimaging Lab, Department of Diagnostic and Interventional Radiology, University Hospital Bonn, Venusberg-Campus 1, Building 7, 53127 Bonn, Germany

**Keywords:** Pain, Neuroscience

## Abstract

The aim of this study was to investigate central pain representations during loading of the periodontium induced by orthodontic and occlusal stress. Nineteen healthy male volunteers (25.7 ± 2.8 years) were tested on two consecutive days: after phenotyping (questionnaires) and determination of warmth (WPT) and heat (HPT) pain thresholds, functional magnetic resonance imaging was performed as event-related paradigm including 36 tooth clenchings of 3 s duration, alternating with rest periods varying between 20–30 s. The task was performed in absence (T1) and 24 h after placement of an elastic separator between the second bicuspid and the first molar on the right side of the lower jaw (T2). No significant changes in WPT and HPT were observed but pain ratings were significantly elevated at T2. Significantly elevated activation at T2, as compared to T1, was found in bilateral sensorimotor cortex, bilateral secondary sensory cortex, supplementary motor area, right rolandic operculum, and bilateral insula. Our data show for the first time in humans that periodontal stimulation, as tested by tooth clenching in the presence of an elastic separator, goes along with specific expressions of pain at behavioral and neuronal network levels. Findings supplement the existing neuroimaging literature on odontogenic pain.

## Introduction

Odontogenic pain can severely impact quality of life^1^ and originates usually from either the dental pulp or the periodontium^2^. Periodontal pain is often perceived as more locally restricted, as compared to the rather diffuse pain sensation stemming from dental pulp pathologies^3^. Common causes are bacterial infections^3^ or excessive mechanical forces following occlusal trauma or orthodontic treatment^3, 4^. Periodontal pain is encountered in 70–95% of patients undergoing orthodontic treatment^5, 6^, manifesting as pressure pain, tension, or soreness^7^, sometimes even causing treatment discontinuation^4^. Typically, orthodontic pain begins 4 h after placement of separators or orthodontic wires, with a maximum on the second day of treatment^5–7^. Usually pain disappears within the following week^7^, but clinical courses are variable. The molecular consequences of mechanical forces exerted on the periodontium show that longer lasting excessive orthodontic forces result in release of "danger associated molecules". These initiate inflammatory responses and consequently pain symptoms via Toll-like receptors^8–10^ and inflammatory reactions^11^ affect nociceptors and sensory nerve fibers located in the periodontal tissue^12^. According to a Cochrane Review, pain originating from the periodontium is difficult to treat^13^. Therefore, pain symptoms may be an indicator of associated pathological processes and, hence, better understanding the effects that orthodontic mechanical forces have in triggering central pain circuits is mandatory.

Functional magnetic resonance imaging (fMRI) allows investigating human pain processing in the central nervous system (CNS). Regarding odontogenic pain, previous fMRI studies have focused on pain originating from the pulp, i.e. using electrical stimulation^14–16^, ice spray stimulation^17^, or natural air^18^. While brain activity during non-painful tactile stimulation of periodontal mechanoreceptors has been studied previously^19^, brain activity related specifically to painful stimulation of the periodontium and the periodontal ligament (PDL) has not been investigated. The PDL is innervated by A delta and C fibers, hence providing the peripheral innervation for transmitting pain and pressure sensations to the CNS^20, 21^. However, there is yet no clear understanding how periodontal pain is represented in the brain.

Here, we investigate for the first-time brain activity stemming from mechanically induced periodontal pain elicited during experimental tooth clenching combined with orthodontic separators. This task-fMRI approach allows directly measuring brain activation as it typically occurs in patients receiving orthodontic treatment and to infer whether this type of pain goes along with typical activation signatures in the human pain matrix. Moreover, we were interested to study how brain activation relates to clinical variables of pain perception and, thereby, to understand whether a pain stimulus originating from the periodontium goes along with similar activations as those previously described for a pain stimulus originating from the dental pulp^15, 22^.

## Methods

### Participants

Exclusively right-handed male subjects between 20 and 40 years of age were recruited, given that sex hormones may influence pain perception^23^. Exclusion criteria were current and/or past (i) neurological diseases, (ii) psychotropic substance abuse/dependence including alcohol/nicotine, (iii) severe psychiatric disorders, as well as periodontal diseases, acute or chronic pain, other severe medical conditions, and MRI contraindications.

Participants were fully informed about the purpose and risks associated with the study design before providing written, informed consent. The study conformed to current guidelines of the University Hospital Bonn and the Declaration of Helsinki and was approved by the Clinical University Bonn Ethics Advisory Committee (289/13).

### Experimental material

A 2.1 mm elastic separator (Dentalastics, DENTAURUM, Ipsringen, Germany) was used to induce pressure forces to the periodontal ligament. This leads to a non-physiological reversible load on the periodontium and creates pain sensations after being placed for a certain time.

### Experimental procedure

Subjects had to attend on two consecutive days: On day one (T1) general (e.g. age, handedness^24^, nicotine dependence^25, 26^, substance consumption) and psychological (State and Trait Anxiety Inventory (STAI; trait version) and Beck Depression Inventory (BDI)^27^) characteristics have been collected. STAI and BDI were acquired to exclude psychological abnormalities.

STAI state, warmth perception (WPT) and heat pain (HPT) threshold were determined on both examination days (T1 and T2), followed by an fMRI examination in which pain was induced by tooth clenching for 3 s. duration. In order to ensure that participants performed similar jaw closures with equal forces and a minimum of opening the jaws, the occlusion was trained before the fMRI scan. Immediately after the task, subjective evaluation of the perceived pain was collected using Visual Analogue Scales (VAS) for pain intensity and discomfort, as well as the McGill-Pain Questionnaire (MPQ)^28^.

**Figure 1:**
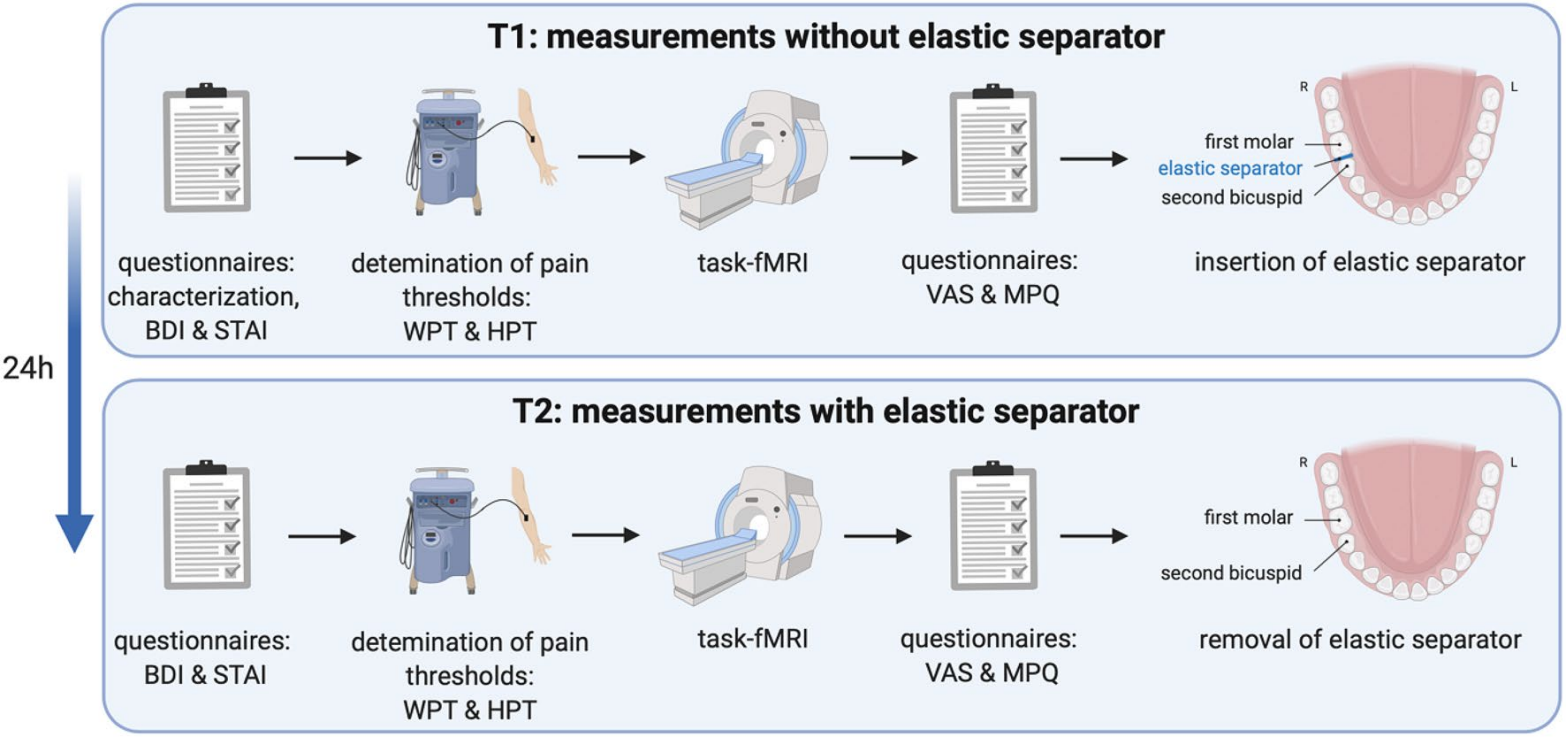
Study design. *L* left, *BDI* Beck’s Depression Inventory, *WPT* warmth perception threshold, *HPT* heat pain threshold, *MPQ* McGill-Pain Questionnaire, *R* right, *STAI* State and Trait Anxiety Inventory, *VAS* Visual Analogue Scale; Figure created with BioRender.com.

The measurement at T1 was carried out as baseline measurement, the measurement at T2 was carried out 24 h after placement (subsequent to T1 examinations) of an elastic separator between the second bicuspid and the first molar on the right side of the lower jaw. Previous studies have shown that a maximum of pain is perceived approximately 24 h after placing a continuous load on a single tooth^6, 7^. The separator was removed subsequent to T2 examinations (Fig. 1).

### STAI and BDI

The STAI consists of two scales (state anxiety, trait anxiety), each containing 20 items, all to be rated on a Likert scale from 1 "not at all" to 4 "very". The BDI measures the severity of depressive symptoms over the past week on 21 categories, each with 4 possible (ranked) answers. The patient is asked to select the most appropriate answer for each category.

### Warmth perception and heat pain threshold determination

Individual sensation and pain perception thresholds were determined using the MEDOC PATHWAY TSA-II NeuroSensory Analyzer (Medoc, Ramat-Yishai, 30095 Israel). The "ascending methods of limits" option with a temperature increase of 0.5 °C per second was chosen, starting at a temperature of 32 °C. A 9 cm^2^ contact thermode was placed at the non-dominant left volar forearm 2 cm below the arm bend. Subjects were instructed to press a button with their right hand as soon as a change in temperature was noticeable (warmth perception threshold, WPT) and as soon as thermal heat perception was accompanied for the first time by a painful sensation (heat pain threshold HPT). Three test trials were followed by 5 main trials that were averaged to determine the respective WPT and HPT.

### fMRI acquisition and paradigm

All MRI examinations were performed at the Department of Diagnostic and Interventional Radiology, University Hospital Bonn, with a 3 T clinical MRI System (Ingenia 5.1.7, Philips Healthcare, Best, The Netherlands), equipped with an 8-channel head coil. Per session 349 T2*-weighted volumes (15:23 min) were acquired with a single shot echo-planar imaging (EPI) sequence. The parameters were as follows: 41 slices acquired in an interleaved order, TR: 2595 ms, TE: 35 ms, flip angle: 90°, orientation: AC-PC, SENSE = 2, matrix: 64 × 64, acquired voxel size: 3.6 × 3.6 × 3.6 mm^3^. For anatomical reference a 3D-T1 weighted sequence (4:39 min) was acquired within each run (slice orientation: sagittal, acquisition matrix: 256 × 256, acquired voxel size: 1 × 1 × 1 mm^3^, sequence type: 3D FFE, TR: 7.6 ms, TE: 3.9 ms, flip angle: 15°).

**Figure 2:**
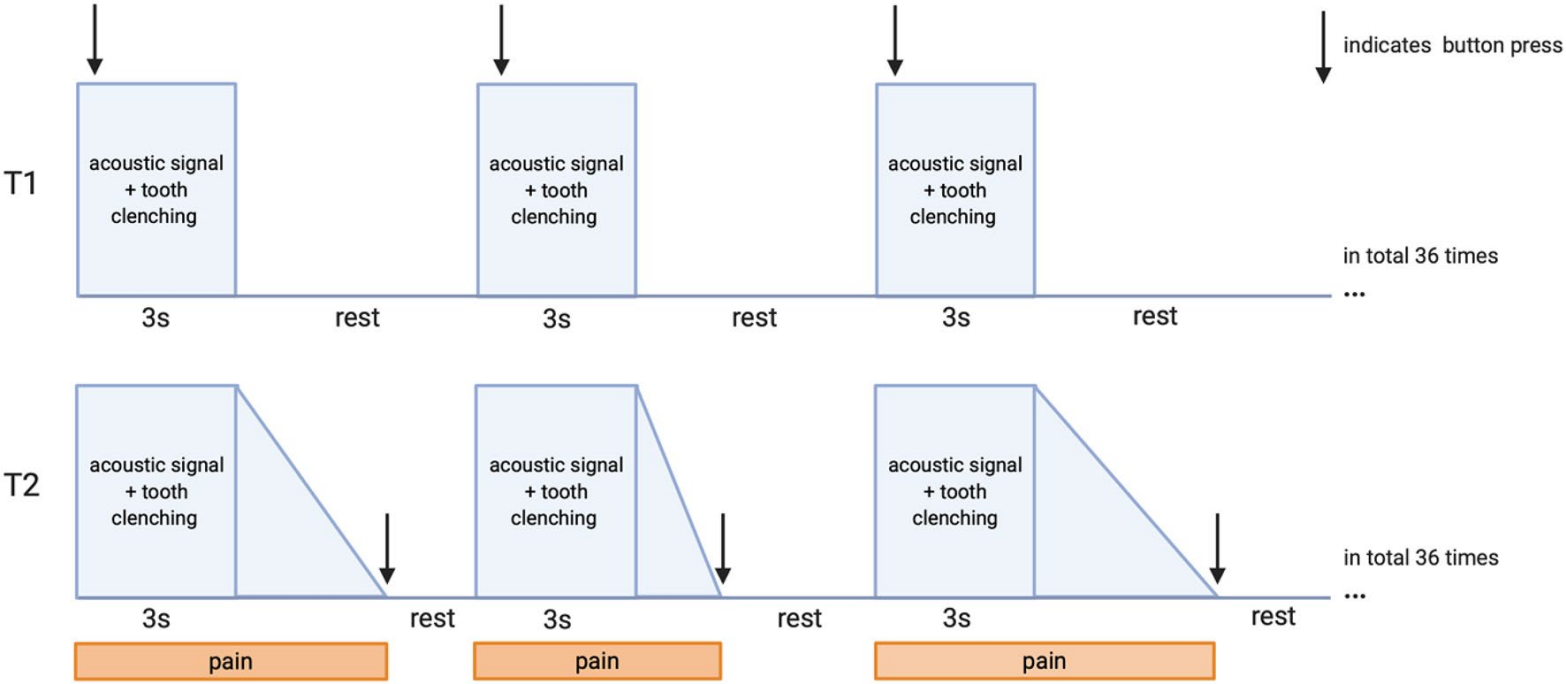
Schematic illustration of the fMRI task at T1 and T2. Figure created with BioRender.com.

Each active condition was signaled by an acoustic sound and occurred 36 times in an event-related design and being. Each static tooth clenching of 3 s duration lasted until the sound stopped. At T1 subjects pressed a button at the beginning of the acoustic sound, at T2 subjects pressed at the end of pain sensation induced by the tooth clenching (defined end of the active condition), allowing to capture the entire pain sensation in the active condition. During the rest intervals (baseline; variable 20–30 s durations), participants were asked to hold the lower jaw in a relaxed position without tooth contact. For schematic illustration of the task see Fig. 2.

### Evaluation of individual pain perception

Immediately after fMRI, MPQ and VAS ratings for anxiety, pain intensity and discomfort were collected (100 mm horizontal lines from “0” meaning ”no pain”/”no anxiety” and “10” meaning ”strongest imaginable pain intensity/discomfort”/”extreme anxiety”).

The MPQ consists of a list of 78 words describing pain. These are grouped in 20 blocks of two to six words each, covering four categories (sensory, affective, evaluative components, others). One word is to be selected per block. Blocks can be skipped if not appropriate. The top word is given a score of 1, the next word a score of 2, and so on and the number of words chosen (NWC) to the total number of words (0–20) is calculated. In addition, the total pain rating index (PRI) (0–78), based on the rank and score of the words in their category was calculated for sensory (PRI-S), affective (PRI-A), evaluative (PRI-E), and other (PRI-M) components. In addition, intensity of perceived pain was rated: 0 = painless, 1 = mild, 2 = uncomfortable, 3 = tormenting, 4 = terrible, 5 = horrible.

### Data analysis

All statistical analyses of behavioral data were performed using SPSS 22 (SPSS Inc., Chicago, Illinois). Criteria for parametric tests were verified by using a Shapiro–Wilk test (testing for normal distribution). Normally distributed data were analyzed using the students paired *t* test, the Wilcoxon signed-rank tests was applied for not normally distributed data. Effect sizes were calculated by Pearson’s correlation coefficient *r*. A value of 0.1 indicates a small effect size, values of ≥ 0.3 a medium effect size, and values ≥ 0.5 a large effect size. Differences were considered significant at p ≤ 0.05. All data are presented as M ± SD, unless stated otherwise.

#### fMRI data analysis

MRI data were analyzed using Statistical Parametric Mapping version 12 (SPM12; Wellcome Centre for Human Neuroimaging, London, UK) implemented in Matlab (The Mathworks Inc., Sherborn, MA, USA). First, a mean T1 of all acquired structural images (of each MRI session) per subject was created using longitudinal registration^29^. The mean T1 image was normalized to the MNI template provided by SPM12. Preprocessing of the functional image series included realignment using the first image as the reference, applying a 5th degree B-Spline interpolation). Further preprocessing steps included slice-time correction and co-registration of the fMRI time series to the derived individual mean T1 data sets. Data were normalized by applying the transformation derived during the normalization step of the mean T1 data set^30^. The interpolation method for the normalization steps was set to 5th degree B-Spline. Finally, the data were spatially smoothed using a 8 mm full width at half maximum Gaussian kernel.

The general linear model was set up as follows: for each event, the individual end points (time when pain sensations returned to baseline) were defined on the T2 data sets. Identical epochs were used for T1 data sets. Thereby, we assured that the identical motor activity (T1 and T2) and the entire pain perception (T2) induced by the tooth clenching was captured. Additionally, the six motion parameters (derived from realignment) were entered as nuisance factors. All sessions (T1 and T2) per subject were entered into one first level model defining separate sessions. Contrasts “tooth clenching T1" and "tooth clenching T2" were transferred to second level analysis.

For the group analysis, a one sample *t* test and a paired *t* test were carried out. As values for the STAI state were significantly different between the two examination days, it was added as covariate of no interest in both models (one sample *t* test and paired *t* test). Only clusters p < 0.05 (FWE-corrected) with a primary cluster-forming threshold of uncorrected p < 0.001 will be reported.

Anatomical localizations of activation peaks were determined using the SPM Anatomy Toolbox version 2.2^31^ and the AAL WFU PickAtlas^32^.

#### Regression analysis

Finally, linear regression analyses were performed in SPM12 between the BOLD signal (whole brain) and the VAS pain (intensity and discomfort), VAS anxiety, MPQ, WPT, and HPT values.

## Results

### Participants

Two of 21 subjects were excluded from our final analysis (N = 1 due to lack of compliance and N = 1 was due to left-handedness). The final sample size was N = 19 participants with a mean age of 25.7 ± 2.8 years. According to the Edinburgh Handedness Inventory, subjects mean laterality quotient was 75.5 ± 14.0, indicating right-handedness.

### STAI and BDI

STAI trait (32.6 ± 9.9) and BDI (3.0 ± 3.9) scores were in a normal range. STAI state revealed a significant difference between examination days (T1: 35.4 ± 11.7; T2: 32.1 ± 12.3, t(18) = 3.7, p = 0.002, r = 0.66).

### Warmth perception and heat pain threshold

Mean WPT was 33.7 ± 0.8 °C at T1 and 33.9 ± 0.9 °C at T2. HPT was on average 43.2 ± 2.8 °C at T1 and 43.6 ± 2.8 °C at T2. None of the thresholds differed significantly between the two examination days (WPT: t(18) = − 0.7, p = 0.473 r = 0.16; HPT: t(18) = − 1.0, p = 0.323, r = 0.23).

### Individual pain perception

#### MPQ

Data of the MPQ were not normally distributed, hence Wilcoxon-Test was used to analyze the data. All pain scores increased from T1 to T2 (see Table 1). Differences between T1 and T2 were significant for all pain scores, except for PRI-A (see Table 1).

**Table 1:** Statistics of McGill Pain Questionnaire for T1 and T2.

	T1	T2	p-value Shapiro–Wilk-Test	Z-value	Asymptotic significance	r
NWC	2.1 ± 6.2	5.0 ± 6.0	0.003	− 3.3	0.001	− 0.54
PRI	4.3 ± 12.9	10.3 ± 13.0	0.010	− 2.9	0.004	− 0.47
PRI-S	1.0 ± 3.0	2.9 ± 3.1	0.003	− 3.3	0.001	− 0.54
PRI-A	0.5 ± 1.6	0.6 ± 1.6	< 0.001	− 1.4	0.157	− 0.23
PRI-E	0.1 ± 0.3	0.5 ± 0.5	< 0.001	− 2.6	0.008	− 0.42
PRI-M	0.4 ± 1.3	1.0 ± 1.3	< 0.001	− 2.6	0.010	− 0.42
Intensity of the perceived pain	0.2 ± 0.5	1.0 ± 0.7	0.019	− 3.0	0.003	− 0.49

Values for T1 and T2 are presented as mean ± standard deviation. *NWC* number of words chosen, *PRI* total pain rating index, *PRI-A* affective pain, *PRI-E* evaluative pain, *PRI-M* other pain, *PRI-S* sensory pain.

#### VAS

Data of the VAS were not normally distributed (Shapiro–Wilk-Test for anxiety: p = 0.001, intensity: p = 0.004, discomfort: p = 0.016), hence Wilcoxon-Test was used to analyze the data. The VAS for anxiety at T1 was 0.3 ± 0.6. At T2 the mean value was 0.4 ± 0.7. There was no significant difference between T1 and T2 (Z = − 0.2, p = 0.874, r = − 0.03).

Evaluation of subjectively experienced pain intensity showed a value of 0.05 ± 0.10 at T1 and 1.05 ± 1.1 at T2. Discomfort of subjectively experienced pain was 0.07 ± 0.13 at T1 and 1.8 ± 1.70 at T2. Both scales showed a significant difference between the two examination days (intensity: z = − 3.4, p = 0.001, r = − 0.55; discomfort: z = − 3.4, p = 0.001, r = − 0.55).

### fMRI

**Figure 3:**
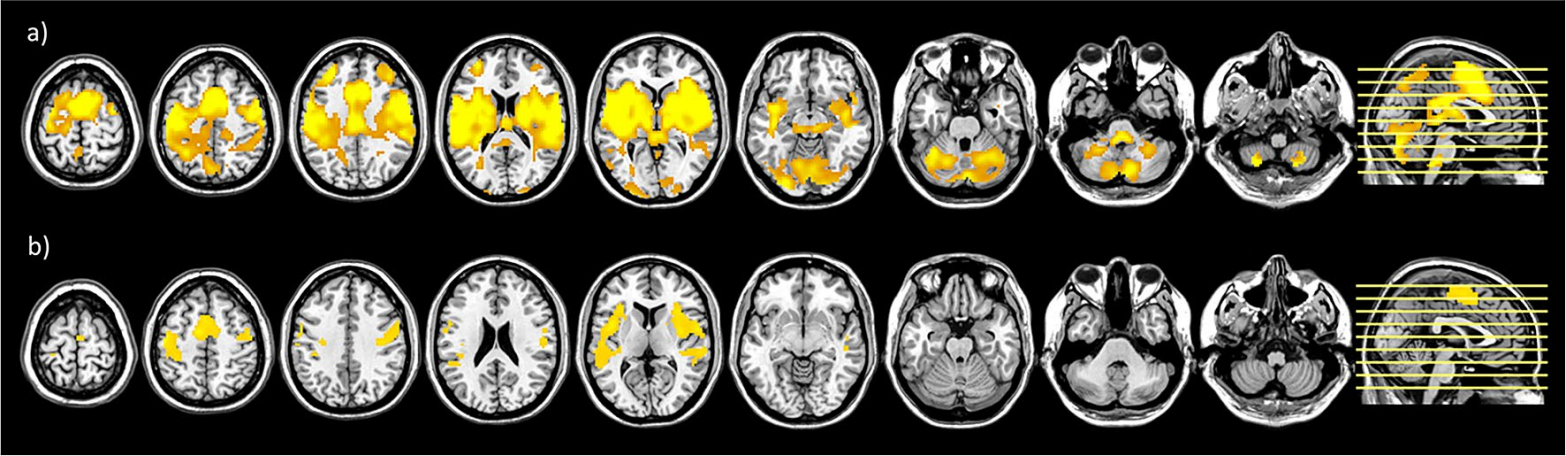
Presentation of activation clusters derived from group-level analysis. (**a**) Activations derived from one-sample *t* test of T2: painful tooth clenching activated bilateral insula (anterior and posterior), bilateral thalamus, bilateral S2, bilateral inferior frontal gyrus, bilateral putamen, bilateral inferior parietal lobule, middle cingulate gyrus, bilateral middle frontal gyrus, bilateral superior frontal gyrus, bilateral cerebellum and left M1; (**b**) Activations derived from the paired *t* test comparing T1 versus T2: painful tooth clenching activated left S1, bilateral S2, bilateral M1, SMA, right rolandic operculum, bilateral insula (anterior and posterior); data are thresholded at p < 0.001, FWE cluster corrected.

The one sample *t* test for the contrast “tooth clenching” at T2 revealed significant activations in brain regions encompassing typical pain processing areas (human pain matrix): bilateral anterior and posterior insula, bilateral thalamus, bilateral secondary somatosensory cortex (S2), bilateral inferior frontal gyrus (IFG), bilateral putamen, bilateral inferior parietal lobule (IPL), middle cingulate gyrus (MCC), bilateral middle frontal gyrus (MFG), bilateral superior frontal gyrus (SFG), bilateral cerebellum and left primary motor cortex (M1) (Fig. 3a). This was not the case at T1. Comparing the BOLD response from T1 with T2 using a paired *t* test revealed stronger activations at T2 in bilateral S1, bilateral S2, bilateral M1, SMA, right rolandic operculum, and bilateral insula (anterior and posterior) (Fig. 3b; Table 2).

**Table 2:** Peak coordinates of the observed significant clusters derived from the paired *t* test T2 > T1.

Brain region	Side	p_FWE_ (cluster corr)	Cluster size	x	y	z
**S1**	L	< 0.001	1109	− 51	− 13	50
Insula	L			− 39	5	11
M1	L			− 42	− 19	59
**SMA**	L	< 0.001	496	− 9	− 4	53
SMA				0	11	50
SMA	R			15	− 4	50
**Insula**	R	< 0.001	1095	36	− 4	11
Rolandic operculum	R			57	− 4	14
M1/S1	R			51	− 7	50

Coordinates (x,y,z) are in MNI space. *L* left, *M1* primary motor cortex, *R* right, *S1* primary somatosensory cortex, *SMA* supplementary motor area.

#### Regression analyses

The regression analyses showed no significant results, neither between the BOLD response and the VAS (anxiety, pain intensity, discomfort) or MPQ, nor between the respective BOLD responses and the WPT or HPT.

## Discussion

Results show that static tooth clenching in the presence of a unilateral elastic separator, as compared to physiological tooth clenching, is associated with increases in perceived pain (MPQ, VAS pain intensity, VAS pain discomfort) that go along with widespread activation increases in typical regions of the human pain matrix. Between conditions, we observed that tooth clenching in the experimental condition was associated with significantly elevated activity in S1, S2, M1, SMA, rolandic operculum, and the insula. While regression analyses did not reveal significant results, findings nevertheless argue for congruent expressions of pain at behavioral and neuronal levels. This first imaging study capturing periodontal pain emphasizes that circumscribed dental manipulations, as constituting routine clinical practice in orthodontics, are capable of eliciting characteristic pain signatures.

Areas of enhanced activation during painful tooth clenching occurred in core areas of the human pain matrix according to meta-analytical analyses^33^, also overlapping with meta-analytical analyses of dental pain data (particularly S1 and insula)^34^. The activation of contralateral S1 and S2 during painful tooth clenching can be interpreted as involvement of the so-called ‘lateral pain system’, known for decoding sensory-discriminative dimensions of pain^35^. Notably, work investigating jaw clenching tasks reported activation changes in a similar location of S1/M1 during adaptation to prosthodontic treatment^36^, however, lacking the exaggerated pain responses found in our study.

Stronger anterior and posterior insular activity was found bilaterally during painful tooth clenching. While the posterior insula is part of the lateral pain system, the anterior insula is part of the mesial pain system and is involved in affective pain processing^35, 37^. Similarly, elevated insular activity was found during ‘amplified pain processing’ in persistent dentoalveolar pain disorder (PDAP) patients (compared to matched controls) induced by dentoalveolar pressure stimulation^38^. In this context, it is interesting that reductions of insular activity associated with decreased pain percepts under orthodontic treatment were reported: in patients with orofacial pain, mandibular splint therapy caused a decrease in left anterior insula activity and the magnitude of the decrease in perceived pain correlates with the magnitude of the decrease in insular activation^39^.

We have to acknowledge that the activations found in this study resemble to a certain degree with those reported upon pain stimulation originating from the dental pulp, including the SMA^22^ and anterior insula^15, 22^. Beyond the reported activations in this study, other studies have also reported the involvement of the cingulate cortex in studies on pulpal pain: Lin et al^34^ reported in their meta-analysis that pulpal electrical stimulation induced activations in the cingulate cortex. One possible reason for us not finding activation differences in the mid cingulate cortex might be the generally low level of experienced pain in our behavioral pain ratings. Indeed, work on painful electrical stimulation of a right human maxillary canine tooth by Brügger et al^14^ had previously tested pain intensity effects by using five graded stimulus strengths (i.e. below, at, and above the individually determined pain thresholds). Authors reported a positive linear correlation with pain intensity for the anterior insula bilaterally, the contralateral (left) anterior mid-cingulate, as well as contralateral (left) pregenual cingulate cortices^14^. While mid-cingulate activation was found at T2 (see Fig. 3a) in our study, both cingulate areas did, however, not show a significant differential activity compared to the control condition which can be most likely attributed to the rather low level of pain intensity. While this hints to generally a similar brain network in tooth pain, future studies will have to be a conducted to directly compare pulpal and periodontal pain within one study design, adjusting for individual levels of pain intensity.

Findings provide novel evidence that painful tooth clenchings go along with elevated pain states. Transfer into clinical practice is warranted, for instance by increasing the awareness to possibly correct either position or size of pain-inducing elastic separators in order to avoid patient suffering and, potentially, secondary harm; given that mechanical prolonged periodontal stress may induce local cell death and long-term irreversible change^11, 12^. In that sense, pain should be considered as a physiological warning sign for avoiding bodily harm. Activation of pain systems even during discrete pain percepts (VAS = 2) is evidence that painful sensations are indeed elicited and should be taken as biological signatures of potentially harmful events. In the same line, customized dental orthotics induced a reduction of pain and associated signs of neural inflammation in temporomandibular joint disorder patients and mechanical treatment reduced abnormal hyper-connected salience networks^40^, which are primarily composed of the anterior insula and dorsal anterior cingulate cortex^41^.

### Limitations

The major limitation to be considered is the use of standard elastic separators, possibly resulting in greater pressure in subjects with crowded teeth. Future studies should standardize tooth load. We also have to acknowledge, that conditions T1 and T2 were not randomized, however, the larger activation seen at T2 argues against a repetition effect as this usually manifests as decreased activation. Finally, one may discuss that pain ratings were assessed as cumulative measures after and not during MRI. While this may induce some inaccuracy, this approach has the advantage of reducing cognitive load and imaging artifacts during fMRI.

The results of this study remain descriptive, as our study design precludes direct comparisons between odontogenic pain originating from the periodontium and the dental pulp. This will be a next step allowing to dissociate specific patterns of activation differentiating the different clinical phenotypes as well as extending studies to female gender.

## Conclusions

This study provides first insights into how periodontal pain is represented in the brain. Although we could not find a direct linear relationship between behavioral and fMRI data, the present data show a change in both variables during tooth clenching in the presence of an elastic separator. The large similarity of the activation patterns reported in studies on pulpal pain and our work support the interpretation of widely shared representations within the human pain matrix, instead of fundamentally different activation patterns between pulpal and periodontal pain. Nevertheless, in order to be able to make any firm conclusions on the specifics and the precise differences between these two forms of tooth pain mechanisms, future studies should be conducted that directly study both form of pain in one experimental setup, adjusting for individual pain intensity levels.

## Data Availability

The datasets generated during the current study are not publicly available due to their containing information that could compromise the privacy of research participants but are available from the corresponding author on reasonable request.
